# Feasibility, reliability, and clinical value of genomic assay on pre-therapeutic biopsy for endocrine receptor-positive HER2-negative early breast cancer

**DOI:** 10.3389/fonc.2026.1892354

**Published:** 2026-08-27

**Authors:** Gilles Houvenaeghel, Maxime Souquet Bressand, Valérie Juhan Duguet, Monique Cohen, Catherine Bouteille, Laura Sabiani, Julien Barrou, Loic Juquel, Véronique Brunel, Olivia Quilichini

**Affiliations:** 1 Department of Gynecology, Hopital Européen, Marseille, France; 2 Department of Medical Oncology, Hopital Européen, Marseille, France; 3 Department of Radiology, Hopital Européen, Marseille, France; 4 Department of Surgical Oncology, Institut Paoli Calmettes, Marseille, France; 5 Department of Gynecology, Hopital de la Conception, Assistance Publique- Hôpitaux de Marseille (AP-HM), Marseille, France; 6 Department of Pathology, Hopital Européen, Marseille, France

**Keywords:** breast cancer, core needle biopsy, genomic assay, neoadjuvant therapy, sentinel lymph node biopsy

## Abstract

Endocrine receptor-positive (ER+) and HER2-negative breast cancer (BC) represents approximately 80% of all BCs. Most patients are treated with upfront surgery; however, 15%–30% will develop late recurrences. Genomic assay indication is usually based on postoperative pathological data including histology subtype, tumor size, lymph node status, SBR grade, and Ki67. Performing genomic testing on core needle biopsy specimens prior to surgery may offer several advantages. In this manuscript, we assess the feasibility, reliability, utility, and potential benefits of such genomic analyses performed on core needle biopsies. Several factors may lead to proposing genomic assay analysis on biopsy: (1) optimization of the patient pathway by reducing time to therapeutic decision-making, (2) predicting response to neoadjuvant chemotherapy (NAC) or neoadjuvant endocrine therapy (NET), and (3) refining prognostic assessment to guide adjuvant chemotherapy decisions in patients for whom axillary surgery is not planned. Given the feasibility and reliability of genomic assay on core needle biopsies, it can be suggested that this practice may become more common in the near future. Knowledge of the evolutive risk determined by the result of genomic assay, as well as clinicopathological characteristics, allows more precise personalization of the therapeutic strategy, including the choice between upfront surgery and neoadjuvant therapy, the selection of systemic treatments, and decision-making in the absence of axillary staging.

## Introduction

Breast cancer (BC) is the most common cancer in women worldwide (1) and in France. Early BC in countries with BC screening accounts for 90% of luminal BC (2–4). The prognosis of luminal endocrine receptor-positive (ER+)/HER2-negative early BC is generally favorable, but 15%–30% will develop metastases with late relapses beyond 5 and 10 years (5). Recurrence rate among luminal high-risk patients with BC may reach 25%–30% (6–8).

Tumor genomic assays (9–13) are commonly used to determine the benefit of adjuvant chemotherapy in ER+/HER2-negative BC, depending on age and in particular menopausal status and on tumor size and lymph node characteristics.

ER+/HER2-negative BC represents approximately 80% of all BCs (14) and is predominantly managed with upfront surgery. The rate of ER+/HER2-negative BC increases with age: 77.8%, 83.0%, 87.0%, and 93.4% for patients 50–59, 60–69, 70–79, and ≥80 years old, respectively, with a parallel decrease in the use of adjuvant chemotherapy administered according to these age groups (42.7%, 35.3%, 24.0%, and 7.5%) (14).

The indication for genomic assay is generally based on postoperative pathological data in the case of upfront surgery including tumor histology, tumor size, lymph node status, SBR grade, and Ki67 level in order to clarify the expected benefit of adjuvant chemotherapy in selected cases. Performing tumor analysis using genomic testing prior to initial surgery, based on biopsy, could offer several advantages and could potentially improve the time to initiation of adjuvant treatment. We evaluate the feasibility, reliability, utility, and potential benefits of such genomic analyses performed on biopsies in a narrative review.

## Methods

Literature search was realized with PubMed from the year 2005 with search terms “early breast cancer genomic assay on core needle biopsy” (12 references), “breast cancer genomic assay on core needle biopsy and neoadjuvant chemotherapy” (16 references), and “breast cancer genomic assay on core needle biopsy and neoadjuvant therapy” (15 references).

## Results

### Feasibility was studied by determining the technical success rate

Jakubowski et al. (15) reported genomic signature analysis on 972,673 samples with technical success rates of 94.5% for biopsy specimens and 97.3% for surgical specimens. Northgraves et al. (16) reported 227 Oncotype DX^®^ analyses performed on biopsies, with a success rate of 98.2% in the PRE-DX study.

### Reliability was assessed by evaluating the concordance of the results on biopsy and surgical specimen

The concordance between paired core biopsy and surgical excision samples for ER, PR, and HER2 receptor status had been reported in several studies. In a meta-analysis of 21 studies (17), concordance rates were 92.8% (*n* = 2,450 pairs) for ER and 85.2% (*n* = 2,448 pairs) for PR. You et al. (18) reported concordance rates of 97.1% (*n* = 1,219 pairs) for ER, 95.0% (*n* = 1,219 pairs) for PR, and 84.6% for HER2. In the Stull et al. study (19), concordance of PR status reached 88%. In contrast, concordance between biopsies and surgical samples were lower for histologic grade, generally under 80% (20–22).

In the Jakubowski et al. study (15), 919,701 samples were successfully analyzed, including 13% of biopsy specimens (*n* = 119,715) and 87% of surgical specimens (*n* = 799,986). ESR1-positive rates were similar between biopsy and excision samples (97.1% vs. 97.9%), with a high concordance in ER status classification (96.8% vs. 97.6%). The distribution of recurrence score (RS) values was similar between biopsies and excision samples with a median (interquartile range) of 16 (10–22) and 16 (11–22), respectively. RS distributions were consistent across age groups (<50 vs. ≥50 years) and nodal status (pN0 vs. pN1mi/pN1). The proportion of RS 0–25 was similar between biopsies and excision samples (82.5% vs. 83.2% in pN0 and 85.1% vs. 86.3% in pN1, respectively).

The study by Stull et al. (19) reported a 92.0% concordance rate for high versus low RS based on 24 biopsy and surgical specimen analyses. Nassar et al. (23) reported data from 134 biopsy and surgical specimen analyses, with a Cohen’s kappa of 0.64 (95% CI, 0.44–0.83). The concordance rate for high versus low RS was 91.8%. Lin’s concordance correlation coefficient for continuous RS values was 0.86 (95% CI, 0.80–0.90).Müller et al. (24) reported results from 40 paired biopsies and excision samples, showing a strong correlation for the EndoPredict (EP) score (Pearson *r* = 0.92) and an excellent agreement for risk classification (95%).

In the Zanetti-Dällenbach et al. study (25), concordance from 22 paired biopsies and surgical sample was 82% (18/22) with RNA analysis.

Nimmi et al. (26) found no significant differences in breast cancer index (BCI) scores between 262 core needle biopsy and 1,292 excision samples, in both node-negative (*n* = 1,152) and node-positive (*n* = 402) patients. Crozier et al. (27) reported a high concordance (90.9%) of MammaPrint results between 121 matched core needle biopsies and surgical resection specimens.

Qi et al. (28) reported a lower concordance rate (74%) in 50 pairs of samples whereas Orozco et al. reported a high concordance rate (91% and 82%) across two independent datasets using Oncotype DX.

Overall, concordance rates across studies ranged from 74% to 95%, with a pooled concordance of 88.97% (347/390 paired samples) (**Table 1**) (19, 23–25, 27, 28). The discordance of approximately 11% may be considered acceptable in relation with tumor heterogeneity especially for large tumors. For multifocal tumors, several core needle biopsies should be performed.

**Table 1:** Concordance of genomic assays between core needle biopsy and surgical specimens.

Author	Concordance (%)	Paired samples (*n*)	Concordant cases (*n*)	Total (*n*)	Genomic assay
Jakubowski	97.1			119,715	RT-PCR
Stull	92.0	24	22	24	Oncotype DX (high vs. low RS)
Nassar	91.8	134	123	134	Oncotype DX (high vs. low RS)
Müller	95.0	40	38	40	EndoPredict (high vs. low risk)
Zanetti-Dällenbach	82.0	21	17	21	RNA-based assay
Crozier	90.9	121	110	121	MammaPrint
Qi	74.0	50	37	50	RT-PCR
**Total**	**88.97**	**390**	**347**	**390**	

Nb, number; RS, recurrent score. Bold values: total results.

### Why test on pre-therapeutic biopsy?

Several factors may lead to proposing genomic assay analysis on biopsy: (1) optimization of the patient pathway by shortening the time to therapeutic decision-making, (2) prediction of response to neoadjuvant chemotherapy (NAC) or neoadjuvant endocrine therapy (NET), and (3) evaluation of prognosis assessment to guide adjuvant chemotherapy decisions in patients for whom axillary surgery is not planned.

Optimizing the patient journey relies on reducing the time for genomic assay results, thereby facilitating earlier treatment decisions and shortening the interval to adjuvant chemotherapy initiation. This approach may also reduce patient anxiety associated with waiting for treatment planning, while helping to meet recommended timelines for chemotherapy initiation, which may impact treatment efficacy.

Indeed, according to the genomic assay test, several steps are required, including shipping, processing, and reporting back, requiring a dedicated and optimized pathway in order to obtain results within a clinically acceptable timeframe for neoadjuvant treatment planning.

The PRE-DX study (16), a prospective, randomized, multicenter British study with genomic assay analysis on biopsy, included 341 patients. The study did not meet its primary endpoint: number of clinical management steps between diagnosis and the proposal of adjuvant treatment. Among the secondary endpoints, an 8-day reduction between surgery and the start of adjuvant treatment was reported, as well as an improvement in patient wellbeing, including lower anxiety and depression scores.

### Genomic assay results obtained from biopsy have made it possible to predict the response to NAC

However, pathologic complete response (pCR) is not a universally validated biomarker for long-term outcomes in ER+/HER2-negative BC, compared to triple-negative (TN) and HER2-positive tumors. Furthermore, the use of genomic assay on biopsy was not recommended in France until the end of 2025, with a strategy opposing their use in France that could, in these cases, represent a therapeutic escalation, but this use has been validated in 2026. It is important to determine whether genomic assays are used in high-risk patients and whether they are more sensitive to NAC than in low-risk patients. However, pCR correlates poorly with survival in luminal tumors, so even if genomic scores predict pCR, the clinical value remains uncertain.

In ER+/HER2-negative BC, data from seven studies reported a low overall pCR rate of 5.4%, but demonstrated that patients with a high RS (>25) had a 4.47-fold higher likelihood of achieving pCR compared to those with a low RS (10.9% vs. 1.1%) (29).

In 2005, Gianni et al. (30) reported, among 82 patients with locally advanced BC treated with neoadjuvant paclitaxel and doxorubicin, that RS was positively associated with the likelihood of pCR (*p* = 0.005), suggesting that patients with a higher risk of recurrence may derive greater benefit from chemotherapy.

Straver et al. (31) reported that none of the good prognosis MammaPrint patients achieved pCR (0/23), whereas 29/144 patients (20%) with a poor-prognosis signature group achieved pCR (*p* = 0.015), including 38 TN tumors within the poor-prognosis group. Authors concluded that tumors with a favorable genomic profile are unlikely to achieve pCR, whereas those with a poor-prognosis signature appear to be more sensitive to chemotherapy.

In a study reported by Mathieu et al. (32) including 94 ER+/HER2-negative BCs with 4.25% pCR, BCI was significantly associated with pCR as a continuous score (*p* = 0.0013) and as categorized risk groups with an odds ratio of 34 for high- vs. low-risk BC (*p* = 0.0055). In multivariate analysis, tumor size and BCI risk groups were significantly associated with breast conservative surgery (*p* = 0.0361 and 0.0102, respectively) with odds ratios of 6.19 (intermediate vs. low risk) and 5.78 (high vs. low risk).

In a study reported by Bertucci et al. (33), the EP predictive value for pCR to NAC was determined from 553 pre-treatment ER+/HER2-negative BCs treated with anthracycline-based NAC. The overall pCR rate was 12%. EP classification was associated with a pCR rate of 7% in the low-risk group (51% of patients at low risk) and 17% in the high-risk group (49% of patients) (*p* < 0.001). EP score remained significantly associated with pCR in multivariate analysis. Authors concluded that EP high-risk ER+/HER2-negative BCs are more likely to respond to anthracycline-based chemotherapy, but the high-risk group was associated with worse disease-free survival (DFS).

In a study reported by Mazo et al. (34), 813 patients, of whom 66 achieved pCR, OncoMasTR, EP, Oncotype DX, and tumor-infiltrating leukocytes were significantly predictive of pCR to NAC in patients with ER+/HER2-negative BC. EP had an odds ratio of 1.76 (95% CI: 1.37 to 2.27) and RS Oncotype DX had an odds ratio of 1.84 (95% CI: 1.44 to 2.35).

Loibl et al. (35) reported, from 428 patients with residual ER+/HER2-negative BC after NAC, that a higher continuous mEPclin score was significantly associated with an increased risk of relapse (HR, 2.16; 95% CI 1.86–2.51; *p* < 0.001) and death (HR, 2.28; 95% CI, 1.90–2.75; *p* < 0.001). Patients classified at high risk by mEPclin showed significantly poorer DFS and overall survival compared with those at low risk. The mEPclin remained significant prognostic for DFS in multivariate analysis (HR, 2.13; 95% CI, 1.73–2.63; *p* < 0.001). The addition of the mEPclin score to a combination of parameters, including CPS-EG score, age, and tumor grade, resulted in a significant improvement of the prognostic power for DFS compared with the clinicopathological model alone. The mEPclin score alone showed higher prognostic values compared with the combinations of clinicopathologic and molecular parameters above.

This approach may allow more accurate risk stratification in patients with residual luminal disease after NAC and help identify those who could benefit from additional post-neoadjuvant therapies, such as CDK4/6 inhibitors.

Dubsky et al. (36) assessed the ability of EP to predict response to NAC. Patients selected for NAC had high-risk EP score (125/134: 93.3%) and 56.0% were diagnosed with Grade 3 BC. Complete response rate (RCB 0-I) to NAC was 24.6%, with better response among patients with high-risk score, with 33 exhibiting RCB 0-I among 125 patients (PPV 26.4%, 95% CI 18.9%–35.0%). EP score significantly predicted treatment response for NAC (AUC 0.736). Authors concluded that EP from diagnostic cores is primarily relevant to tumor response to NAC and EP score from residual tumor in combination with ypT and ypN can identify patients at residual high risk.

Soliman et al. (37) reported that from 764 ER+/HER2-negative BC samples including 59 patients with pCR, there is a significant prediction of pCR by EP and Oncotype DX assays with higher expression scores. Authors concluded that these gene expression scores are predictive of pCR and could therefore inform pre-surgical treatment decisions.

Prat et al. (38) reported that the PAM50 intrinsic subtype was independently associated with pCR in ER+/HER2-negative BC. The pCR rates were 6% and 16% for the luminal A (*n* = 239) and luminal B (*n* = 143) subtypes, respectively. Moreover, within patients that do not achieve a pCR, the ROR-P predictor can identify a group of patients with clinically node-negative disease with an excellent survival outcome at 5 years. High 5-year disease recurrence-free survival (DRFS) rates were observed in patients with ROR-P low disease that did not achieve a pCR with a 5-year DRFS of 92.0% (95% CI, 85.5%–99.1%) in all patients and of 97.4% (95% CI, 92.6%–100.0%) in node-negative BC. The intrinsic subtypes predict pCR, and their predictive value was independent of standard clinicopathological variables.

In 2016, Prat et al. (39) reported that the Prosigna assay pass rate of core needle biopsy was 98.3% (234/238) and 95.8% (207/216) in the chemo-prediction evaluation. The accurate Prosigna assay results can be reliably obtained from diagnostic core needle biopsy, with a high correlation between core needle biopsy and surgical resection specimen from 30 paired samples with a correlation coefficient of 0.90 (range, 0.90–0.98) for ROR score and the four-subtype correlation. Authors concluded that Prosigna ROR score and intrinsic subtype are strong predictors of response to NAC and that the continuous ROR score was a significant predictor of response to NAC (*p* = 0.047).

Callari et al. (40), in a study including 357 patients treated with NAC, reported that the high-proliferation/low-ER group of ER+/HER2-negative BC had a significantly higher pCR rate [OR, 5.01 (1.76–17.99), *p* = 0.005], but poorer outcome [HR = 3.73 (1.63–8.51), *p* = 0.0018] than the low-proliferation/high-ER group. The high-risk group showed the poorest prognosis despite chemotherapy administration [74.1% 5-year disease-free metastasis survival (DMFS), HR = 3.73 (1.63–8.51), *p* = 0.0018]. The low-risk group demonstrated an excellent prognosis even in tumors with residual disease after NAC (96.1% 5-year DMFS), although the high-risk group had poor prognosis (69.2% 5-year DMFS, *p* = 0.01).

Cottu et al. (41) reported phase II results after Prosigna analysis on core biopsy including patients who are not candidates for breast conserving surgery (BCS) treated by neoadjuvant therapy, Letrozole and Palbociclib, or chemotherapy (53 in each arm). RCB0-I rate was 15.7% after NAC and BCS rate was 69% for 9 intermediate ROR risk and for 44 high ROR risk.

Boland et al. (29) reported a systematic review and meta-analysis of the value of a 21-gene expression assay on core biopsy to predict NAC response in estrogen receptor-positive HER2-negative BC. Seven studies (42–48), including 777 patients (44.6%) with a high RS and 967 (55.4%) with a low–intermediate RS, were analyzed. The pCR rate was 5.4% (94 patients) higher in the group with a high RS (74/777) than in the group with a low–intermediate RS (20/967) (10.9 versus 1.1%, risk ratio 4.47, *p* < 0.001).

Recently, Ma et al. (49) reported that high-risk MammaPrint BC ≥ 31 achieved a significantly higher pCR rate than lower-risk BC.

However, other characteristics may help to determine pCR to NAC; for example, patients with PIK3CA mutations are less likely to respond to NAC.

Overall, pCR rates were consistently low in ER+/HER2-negative BC but significantly higher in patients with high genomic risk compared to those with low or intermediate risk (**Table 2**). The pooled pCR rate was 3.88% in low- and intermediate-risk tumors (range: 0%–10.7%; 160/4,126), compared to 13.2% in high-risk tumors (range: 0%–26.4%; 460/3,476) (30–34, 36–38, 40, 42–48, 50, 51, 53–63).

**Table 2:** Literature results of pCR rate to neoadjuvant chemotherapy according to genomic assay level.

Author (year)	Assay	RS groups	Patients (*n*)	pCR (%)	*p*-value
Gianni (2005) (30)	Oncotype DX	RS continuous	89	12.4	0.005*
Mina (2006) (50)	Oncotype DX	Not specified	45	13.3	0.67*
Chang (2008) (51)	Oncotype DX	<18/>30	8/42	0/21.4	0.008
Straver (2010) (31)	MammaPrint	Low/High	23/106	0/15	0.07
Mathieu (2012) (32)	BCI	Low/Int/High	50/31/13	0/9.7/7.7	0.0492
Krijgsman (2012) (52)	MammaPrint	Low/High	29/53	3/11	NR
Gluck (2013) (53)	MammaPrint	Low/High	90/154	6/10	NR
Zelnak (2013) (42)	Oncotype DX	Low-Int/High	11/17	0/17.6	
Bertucci (2014) (33)	EndoPredict	Low/High	282/271	7/17	<0.001
Whitworth (2014) (54)	MammaPrint	LumA/LumB	36/115	3/7	
Bayraktar (2014) (55)	MammaPrint	Low/High	15/44	7/5	NR
Yardley (2015) (45)	Oncotype DX	<18/18–30/>30	19/17/24	0/0/17	0.030
Pivot (2015) (47)	Oncotype DX	<18/18–30/>30	29/28/24	0/6.2/8.6	0.004
Prat (2015) (38)	Prosigna	LumA/LumB	239/143	6/16	
Callari (2015) (40)	Metagene	Low/High	NA	4.4/18.9	0.005
Soran (2016) (48)	Oncotype DX	<18/18–30/>30	27/10/23	0/0/0	
Iwamoto (2016) (56)	Oncotype DX	<18/18–30/>30	751/384/238	4.6/5.7/16.5	Ref/0.983/0.073
Bear (2017) (43)	Oncotype DX	Low/High	14/11	0/14.3	
Whitworth (2017) (57)	MammaPrint	Low/High	95/310	2.1/13	<0.001*
Pease (2019) (58)	Oncotype DX	<18/18–30/>30	227/450/312	2.2/1.6/9.6	<0.001
Kantor (2019) (46)	Oncotype DX	<11/11–25/>25	160/612/605	4.4/1.3/7.8	<0.01
Thekkekara (2019) (44)	Oncotype DX	≤25/>25	40/70	0/16	NR
Nunes (2019) (59)	MammaPrint	Low/High	120/235	2/13	0.0015
Dubsky (2020) (36)	EndoPredict	High only	125	26.4	
Mazo (2020) (34)	Oncotype DX	Not specified	813	8.1	
Soliman (2020) (37)	Oncotype DX + EP	Not specified	764	7.7	0.0041*
Kuemmel (2020) (60)	Oncotype DX	≤25/>25	153/423	7.2/16.1	0.006
Murillo (2020) (61)	Oncotype DX	11–25/>25	20/40	0/12	0.005°
Pardo (2021) (62)	Oncotype DX	<18/18–30/>30	56/62/40	10.7/9.7/27.5	0.0268
Sella (2021) (63)	Oncotype DX	≤25/>25	38/38	5.3/21.1	0.01*

pCR, pathologic complete response; Nb, number; BC, breast cancer; RS, recurrent score. *continuous value, °multivariate analysis, °°axillary pCR.

When stratified by assay, pCR rates remained consistently higher in high-risk groups across all platforms. With Oncotype DX, pCR rates were 3.56% (111/3,116) in low/intermediate-risk tumors versus 12.53% (239/1,907) in high-risk tumors. With MammaPrint, pCR rates were 2.94% (range: 0%–7%; 12/408) in low-risk tumors and 11.6% (range: 5%–15%; 118/1,017) in high-risk tumors. Similarly, with EP, pCR rates were 7% (20/282) in low-risk tumors and 19.9% (range: 17%–26.4%; 79/396) in high-risk tumors.

These pCR rates and clinical response (cR) make it possible to offer surgical de-escalation and a higher rate of BCS.

### What is its utility?

The pCR rate after NAC remains modest for ER+/HER2-negative BC compared to TN and HER2-positive BC with rates exceeding 40%–50%. pCR rates were significantly increased after NAC combined with pembrolizumab for TNBC (Keynote 522) and after NAC combined with anti-HER2 therapies (trastuzumab ± pertuzumab) or combined with antibody–drug conjugate therapy (trastuzumab deruxtecan). Moreover, pCR rates were significantly higher for ER+/HER2-negative BC with a HER2 score of 0 compared to HER2-low (score 1 or 2) (64).

NAC is usually indicated for ER+/HER2-negative BC in order to achieve a reduction in tumor size to allow for BCS if mastectomy is indicated and initial surgery was planned, or to allow for total mastectomy under better conditions for locally advanced BC. Genomic assay results on biopsy help in selecting patients for NAC. For high-risk BC, pCR rates or a good response without pCR supports the use of NAC to enable BCS, primarily in cases of unifocal tumors and in well-selected cases of bifocal or bicentric tumors. Conversely, very low pCR rates or good response rates expected in low-risk BC may argue for primary surgery rather than surgery after NAC.

Genomic assay results on biopsy allow for the prediction and evaluation of the response to NET.

pCR to NET remains low (<10%) for estrogen receptor-positive BC (65). However, compared with NAC, NET using aromatase inhibitors had a similar cR rate and BCS rate, with lower toxicity. Moreover, aromatase inhibitors were associated with a significantly higher cR rate and BCS rate compared with tamoxifen (65).

Across studies, pCR rates remained low but were higher in high-risk genomic groups compared to low/intermediate-risk groups, with pooled rates of 5.5% (21/383) and 11.2% (74/660), respectively (31, 36, 42, 43, 52–55, 57, 59, 66–71). When stratified by assay, pCR rates were extremely low with Oncotype DX (0% in low/intermediate-risk vs. 3% in high-risk tumors), whereas MammaPrint showed pCR rates of 3.2% and 11.8% in low- and high-risk groups, respectively. Results with EP were more heterogeneous, with pCR rates of 27.3% in low-risk and 7.7% in high-risk tumors, likely reflecting small sample sizes and selection bias.

In contrast, cR rates were substantially higher, particularly in low-risk tumors, with overall rates of 54.7% in low/intermediate-risk groups versus 26.9% in high-risk groups. This trend was consistent across assays, including Oncotype DX (53% vs. 22.9%) and MammaPrint (72% vs. 56.5%) (**Table 3**).

**Table 3:** Literature results of pCR or cR rates to neoadjuvant endocrine therapy according to genomic assay level.

Author (year)	Endocrine therapy	Assay	RS groups	Patients (*n*)	pCR	% cR	*p*-value
Akashi-Tanaka (2009) (67)	AIs/Tamoxifen	Oncotype DX	<18/18–30/>30	11/16/16		64/31/31	0.11
Straver (2010) (31)	–	MammaPrint	Low/High	23/106	0/15		0.07
Krijgsman (2012) (52)	-	MammaPrint	Low/High	29/53	3/11		
Gluck (2013) (53)	–	MammaPrint	Low/High	90/154	6/10		
Zelnak (2013) (42)	-	Oncotype DX	<11/11–25	NA	0/0		
Ueno (2014) (68)	Exemestane	Oncotype DX	<18/18–30/>30	32/17/15		59.4/58.8/20	0.042*
Bayraktar (2014) (55)	-	MammaPrint	Low/High	15/44	7/5		
Whitworth (2014) (54)	AIs/Tamoxifen	MammaPrint	LumA/LumB	15/5		80/20	
Whitworth (2017) (57)	AIs/Tamoxifen	MammaPrint	Low/High	35/18		68.6/66.7	
Bear (2017) (43)	Tamoxifen/AIs	Oncotype DX	<11/11–25	12/18	0/0		NS
Iwata (2018) (66)	-	Oncotype DX	<18/18–30/>30	157/84/54		55/42/22	<0.001
Nunes (2019) (59)	–	MammaPrint	Low/High	120/235	2/13		0.0015
Abu-Khalaf (2019) (71)	Exemestane/AIs	-	Low–Int/High	11/16		6.4/25	
Dubsky (2020) (36)	–	EndoPredict	Low/High	44/39	27.3/7.7		
Davey (2021) (69)	Tamoxifen/AIs	Oncotype DX	<18/18–30/>30	32/17/287	0/0/3.1		
Guerrero-Zotano (2024) (70)	Letrozole+Palbo	Oncotype DX	18–25/26–100	32/29	0/3		0.32

pCR, pathologic complete response; cR, clinical response; Nb, number; BC, breast cancer; RS, recurrent score; AIs, aromatase inhibitors. *continuous value, °Davey et al. study: including Bear, Ueno, Iwata, and Akashi-Tanaka studies.

Dubsky et al. (36) reported that the RCB 0-I rate after NET in the low-risk EP group was 27% in comparison with 7.7% in high-risk patients and concluded that the EP result was able to identify patients with poor NET response.

Davey et al. (69) reported a meta-analysis including eight studies and 691 patients treated with endocrine therapy (tamoxifen, aromatase inhibitors, or fulvestrant) using Oncotype DX. Only 22 patients achieved pCR (2.8%), and RS was not associated with pCR. However, low RS was significantly associated with partial response, with higher response rates observed in patients with RS <25 (OR 4.60, *p* < 0.001) and RS < 30 (OR 3.40, *p* < 0.001) compared to higher-RS groups. Additionally, low RS was significantly associated with higher rates of BCS after NET, with 111/189 patients (RS < 18) undergoing BCS compared to 6/17 (RS 18–30) (*p* < 0.001).

The efficacy of NET with Letrozole and Palbociclib was not dependent on pre-therapeutic Oncotype DX RS > 18. Objective response rates were 78.1% (25/32) and 63.6% (21/33) for RS 18–25 and >25, respectively. After NET, two among four patients with RS 18–25 and two among six patients with RS > 25, who initially were candidates for mastectomy, may have BCS (70).

In the Cottu et al. trial (41), after Prosigna analysis on core biopsy, 53 patients (7 intermediate and 46 high ROR risk) who were not candidates for BCS were treated with Letrozole and Palbociclib. RCB 0-I rate was 7.7% and BCS rate was 69%.

Moreover, Prat et al. (72) reported that some patients with luminal B subtype with high-risk ROR disease could achieve molecular downstaging with Ribociclib and Letrozole.

### Which endocrine therapy?

NET during 3 months for elderly patients with locally advanced or early BC has been shown to be more effective with aromatase inhibitors than tamoxifen (73).

In a phase II randomized trial (74), the three aromatase inhibitors, exemestane, letrozole, or anastrozole, were biologically equivalent. Clinical complete response was 20.3% (76/374) and clinical partial response was 48.7% (182/374) with an overall cR of 69.0% (258/374). BCS rate was 68.5% (241/352): 83.0% (157/189) for initial marginal indication for breast conservation, 50.9% (81/159) for patients who were candidates for mastectomy only, and 75.0% (3/4) for patients initially inoperable by standard mastectomy. Both luminal A and B tumors determined with Prosigna assay were highly endocrine-therapy responsive at the clinical and biomarker level. The 5-year cumulative incidence of loco-regional recurrence after BCS was low (1.53%) (75).

Nitz et al. (12) reported that guiding systemic treatment by both RS Oncotype DX and NET response is feasible in clinical routine, with 78.1% response rate for postmenopausal patients with aromatase inhibitors. Endocrine therapy response rate was 78.8% in RS 0–11, 62.2% in RS 12–25, and 32.7% in RS > 25 (*n* = 4,203, *p* < 0.001).

Combination therapy with CDK 4/6 inhibitors and aromatase inhibitors increased efficacy and toxicity compared to endocrine monotherapy with similar efficacy and better safety than NAC (76).

### Utility to predict response to neoadjuvant therapy

Response prediction can be useful when neoadjuvant therapy is offered to achieve tumor shrinkage, making BCS a viable option in situations where a total mastectomy would be necessary as the initial surgery. BCS rates were over 60% in most studies (41, 43, 69, 74). This cR and or pCR prediction, optimized by a genomic test prior to the initiation of treatment, appears useful for making decisions regarding neoadjuvant therapy.

Cao et al. (77) reported breast and axillary response and survival after NAC (14.025 patients) or NET (5804) in women ≥ 50 years with cT2–4 ER+ BC. Tumor downstaging was achieved in 58% of the patients with NAC and 40.5% of the patients with NET, and in-breast pCR was achieved for 9.3% of the NAC and 1.3% of the NET patients. Approximately half of the mastectomy was avoided for the patients with in-breast pCR: 53.6% of the NAC and 43.8% of the NET patients. For patients with clinically involved axillary nodes, nodal downstaging was 29% after NAC and 18.3% after NET, and nodal pCR was 20.3% after NAC and 13.5% after NET. Authors concluded that NET can be used to de-escalate surgery for patients who cannot tolerate NAC or when chemotherapy may not be effective based on genomic assay.

Very low predicted complete response rates could lead to either not performing neoadjuvant treatment or making a choice between NAC or NET. However, cR rates were high after NET, 26.9% to 54.7% according to low/intermediate or high risk in several studies allowing for BCS, and 48.1% and 11.1% for genomic low/intermediate and high risk, respectively (54, 57, 66–68, 71). Han et al. (78) recommend genomic assay to guide the choice between NAC and NET to improve BCS. However, cR rate is not considered as a valid surrogate for long-term outcome in this setting of NET.

Evaluation of the prognosis and the indication for adjuvant chemotherapy when no axillary lymph node surgery is envisaged.

Omitting axillary surgery is increasingly considered in selected patients, based on the results of the SOUND and INSEMA trials in postmenopausal patients with ER+/HER2-negative, Grade 1 or 2, cN0 BC without axillary lymphadenopathy on ultrasound, treated with BCS (79, 80). Eligibility criteria included cT1 tumors in SOUND and cT1–2 tumors in INSEMA. However, omission of axillary staging may raise concerns about potential under-treatment, as occult nodal involvement may be present in approximately 15% of cases, depending on tumor size, grade, and lymphovascular invasion (81). Importantly, the presence of limited nodal involvement (one to three positive nodes) does not systematically indicate the need for adjuvant chemotherapy. Genomic assay allows for a more precise indication for adjuvant chemotherapy based on the risk of disease progression and the expected benefit, for patients with or without lymph node involvement.

Adjuvant chemotherapy rates were 12.9% and 10.4% in the INSEMA trial and 20.1% and 17.5% in the SOUND trial for sentinel lymph node biopsy (SLNB) and no axillary surgery, respectively. Higher rates of adjuvant chemotherapy in the INSEMA trial including greater tumor sizes highlight differences in clinical practice.

However, the rate of patients whose indication for adjuvant treatment depending on the SLNB result is low: 14% of pN0 cases with an RS > 25 and 13% of pN1 cases. The rate of additional adjuvant chemotherapy was estimated at 1% in cases of SLNB compared to the absence of SLNB in patients with an RS > 25 (82). Moreover, low MammaPrint signature risk did not predict a low pN+ rate (*p* = 0.377) in a study (83) that included 668 patients with 72% low-risk BC (481) and 28% high-risk BC (187). Among pN0, 73.3% were low risk, and among pN+, 69.5% were low risk. Fang et al. (84) reported among 238 patients with MammaPrint assay on core needle biopsies—70% low-risk and 30% high-risk patients—that 81% were pN0 and 19% were pN+. The MammaPrint score did not correlate with pathologic nodal stage (*p* = 0.52) and the rate of high nodal burden (pN2) was very low (*n* = 1, 0.5%).

Therefore, performing a genomic test evaluation on biopsy (85) is likely to avoid under-evaluation and under-treatment of adjuvant chemotherapy and to better identify patients in whom any axillary surgical procedure can be avoided without compromising the indication for adjuvant chemotherapy, particularly for cT2 tumors.

## Conclusion

**Figure 1:**
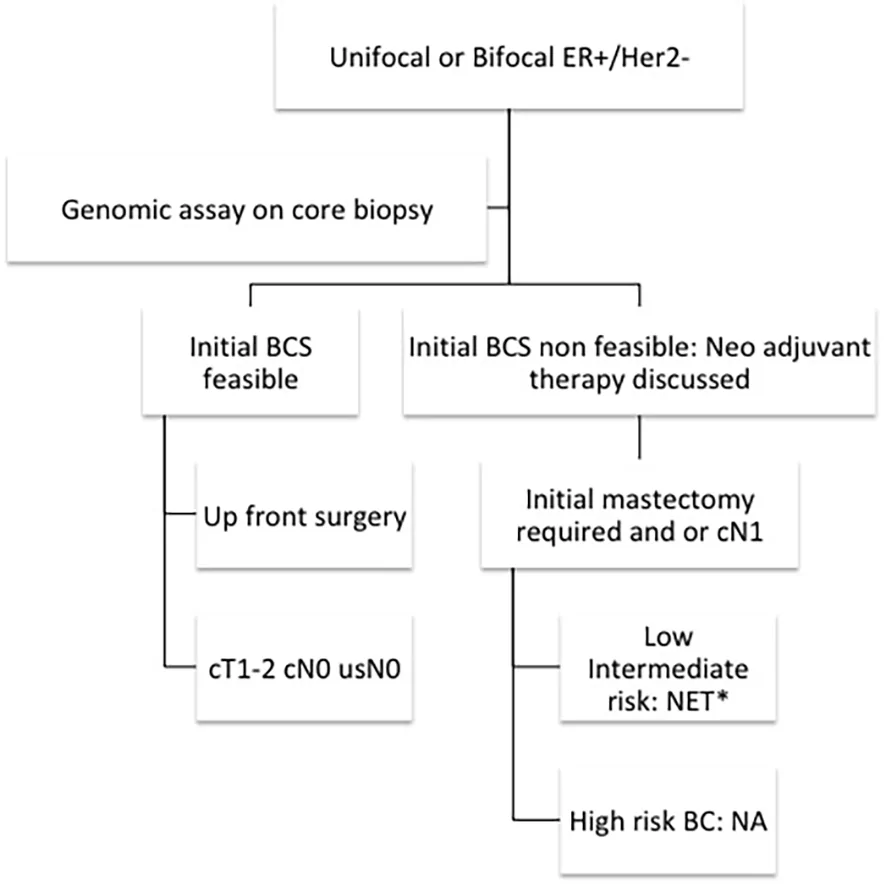
Algorithm incorporating the objective of genomic assay on core biopsy.

Given the encouraging evidence supporting the feasibility and reliability of genomic assays performed on core needle biopsy specimens, this approach is likely to become increasingly integrated into routine clinical practice in the near future. An algorithm incorporating the objective of genomic assay on core biopsy as well as the most relevant indications is presented in **Figure 1**.

Early assessment of genomic risk, combined with clinical and pathological factors such as age, menopausal status, and tumor characteristics, enables a more precise and personalized therapeutic strategy. This includes the choice between upfront surgery and neoadjuvant therapy, the selection of neoadjuvant modality (NAC or NET), and the optimization of adjuvant systemic treatment decisions, particularly in the absence of axillary staging.

However, some limitations must be highlighted, including an approximately 11% discrepancy between the genomic assay results on biopsy and on surgical samples, the uncertain clinical value of pCR for luminal tumors after NAC and the lack of validation of the cR rate as a reliable surrogate for long-term outcomes following NET. Furthermore, the role of genomic assay when omitting axillary surgery requires further study.

Overall, genomic testing on biopsy specimens may represent a key tool to refine treatment selection, support therapeutic de-escalation, and improve patient-tailored care in ER+/HER2-negative BC.
